# Altitudinal pattern of shrub biomass allocation in Southwest China

**DOI:** 10.1371/journal.pone.0240861

**Published:** 2020-10-22

**Authors:** Mei Liu, Dandan Li, Jun Hu, Dongyan Liu, Zhiliang Ma, Xinying Cheng, Chunzhang Zhao, Qing Liu

**Affiliations:** 1 Key Laboratory of Mountain Ecological Restoration and Bioresource Utilization & Ecological Restoration Biodiversity Conservation Key Laboratory of Sichuan Province, Chengdu Institute of Biology, Chinese Academy of Sciences, Chengdu, China; 2 College of Life Sciences, Sichuan University, Chengdu, China; 3 Ecological Security and Protection Key Laboratory of Sichuan Province, Mianyang Normal University, Mianyang, China; 4 University of Chinese Academy of Sciences, Beijing, China; 5 College of Life Sciences, China West Normal University, Nanchong, China; 6 Chengdu University of Technology Institute of Ecological Environment, Chengdu, China; Sichuan Agricultural University, CHINA

## Abstract

Shrubs play an important role in the global carbon cycle and are particularly sensitive to climate change. However, the altitudinal pattern of biomass allocation in mountainous shrubs and its responses to climate change are still unclear. In this study, biomass accumulation and allocation of the shrub community and their relationships with climatic factors were investigated in 331 sampling sites along an extensive altitudinal gradient (311–4911 m) in Southwest China. The results showed that the above-ground biomass (AGB) and the total biomass (TB) of the shrub community decreased quadratically (R^2^ = 0.107) and linearly (R^2^ = 0.024) from 9.86 to 0.15 kg·m^-2^ and 15.61 to 0.26 kg·m^-2^ with increasing altitude, respectively. However, the below-ground biomass (BGB) and TB of the herb layer increased quadratically with increasing altitudes (R^2^ = 0.136 and 0.122, respectively. *P*<0.001). The root/shoot ratio (R/S) of the community and its component synusiae increased gradually with increasing altitudes (*P*<0.001). The standardized major axis (SMA) indicated an isometric relationship between AGB and BGB for the whole shrub community, but allometric relationships were found for the shrub and herb layer. Redundancy analysis and Pearson correlation analysis showed that the biomass and R/S were significantly correlated with mean annual temperature (MAT), mean annual precipitation (MAP) and reconnaissance drought index (RDI). These findings indicate that shrub biomass allocation is strongly affected by the altitude, MAT and MAP and support the isometric relationship of AGB and BGB partitioning at the community level on mountainous shrub biomes.

## Introduction

Biomass accumulation and allocation are important to quantify the carbon stocks and fluxes within the global carbon cycle [1, 2]. Above- and below-ground biomass allocation affects the overall functions of the biogeochemical cycles and ecosystem and is thus a central issue in plant ecology [2, 3]. Changes in biomass allocation are important for plants to adapt to heterogeneous habitats and as an indicator for alterations in habitat environment [4]. The root/shoot ratio (R/S) indicates the differential investment of photosynthates between the above- and below-ground organs; the R/S is influenced by soil types, phenology, environmental factors, etc. [5–7]. Climatic factors (such as temperature, precipitation and moisture) are usually reported to affect the plant biomass allocation, especially the partitioning of vegetative and reproductive biomass [8–10]. Temperature drives the global patterns of forest biomass allocation in roots, stems, and leaves [10]. Mean annual temperature (MAT) influences the spatial patterns of above-ground biomass (AGB) in alpine meadows on the Tibetan Plateau [11]. Precipitation is the most important determinant factor in shaping the AGB and below-ground biomass (BGB) distribution patterns in many ecosystems [2, 12]. The responses of biomass accumulation and allocation to mean annual precipitation (MAP) in China’s forests are approximated by a cubic and quadratic relationship [9]. The AGB abundance in alpine steppe was positively correlated with MAP [11]. It is also reported that AGB and BGB on the alpine steppe in northern Tibet would decrease because of aridity in the future [2].

Shrub ecosystems are widely distributed from the tropics to polar regions [13, 14]. Shrublands play important roles in global C and N storage, succession procedures, regional eco-environmental protection, improvement in water quality, preservation of biodiversity and soil conservation [13, 15, 16]. The total shrub-covered area in China is 2×10^6^ km^2^, approximately one-fifth of the total land area [16]. Moreover, shrub ecosystems are the main carbon pool (6.69 ± 0.32 Pg C) in China [16, 17]. However, shrubs are often considered as a successional stage of plant communities or a part of grassland and forest ecosystems [18]. Previous studies on biomass allocation of shrubs focused on specific species at the individual level [13, 19, 20]. Community-level biomass accumulation and allocation, and their relationships with climatic factors in shrub ecosystems, have remained unclear [21].

Isometric allocation and optimal partitioning are two important hypotheses about the biomass partitioning between roots and shoots of plants. These concepts are powerful quantitative tools to predict plant structures and functions as well as the intraspecific and interspecific relationships among plant organ biomass at the individual, community or ecosystem level [22, 23]. Recently, many researches have focused on biomass distribution patterns at the individual and community levels in forestlands and grasslands, and the relationships between roots and shoots were different in various study areas [3, 24–29]. At the community level, the relationships between log AGB and log BGB in forests and grassland support the isometric hypothesis [3, 25, 29]; however, it remains unknown whether this hypothesis is supported in shrublands.

Altitude determines the variation in environmental factors and plant community composition [30]. Altitudinal gradients are powerful ‘natural experiments’ for testing evolutionary and ecological responses of biota to geophysical influences in mountainous regions [30, 31]. Biomass allocation strategies along altitudes have attracted extensive attention [28]. For example, 54 populations of *Picea abies* (L.) Karst. at eight altitudes (600–1500 m) showed nonlinear responses of biomass to elevation in southern Poland [32]. The AGB of 57 perennial herbaceous species decreased with increasing altitude (at subalpine 3700 m, alpine 4300 m and subnival≥5000 m altitudes) [28]. The AGB in natural alpine grassland showed unimodal patterns along an altitude gradient (3862–4450 m) in the source region of three rivers on the Qinghai-Tibetan Plateau [33]. The R/S ratio increased linearly with increasing altitude (2880–4040 m) and was significantly correlated with the climate on non-degraded alpine meadow on the northeast Tibetan Plateau [34]. Over the past several decades, researchers have focused on the relationships among climatic factors and altitude with the biomass allocation of forests and grasslands. However, reports about community-level biomass allocation, including its different synusiae (i.e., shrub layer and herb layer) of shrub ecosystems along large range of elevations, are very rare.

Southwest China, including Xizang, Qinghai, Sichuan, Yunnan and Guizhou provinces, is a special eco-geographic region and the core of the ecological security barrier of Tibet. Furthermore, this region, with an extensive altitudinal gradient (72–8848 m), is a biodiversity hotspot and is considered by Conservation International (CI) to be a primary area of strategic resources. Mountainous shrubs in Southwest China account for 52.1% of China’s total shrub compositional types (i.e., evergreen broad-leaved, deciduous broad-leaved, evergreen needle-leaved, evergreen leathery-leaved and desert shrubs), and differ from the northern temperate and southern subtropical shrubs in China [35]. The broad altitude gradient of shrub distributions (300–5000 m) provides ideal conditions for investigating the altitude pattern of shrub biomass allocation and to test its relationship with climatic factors. Therefore, the objectives of this study were to (i) characterize the altitude pattern of biomass allocation in the mountainous shrublands, (ii) test whether the isometric relationship is supported by shrublands at the community level, and (iii) determine the relationship between climatic factors and biomass accumulation and allocation in mountainous shrubs in Southwest China.

## Materials and methods

### Study area

**Fig 1:**
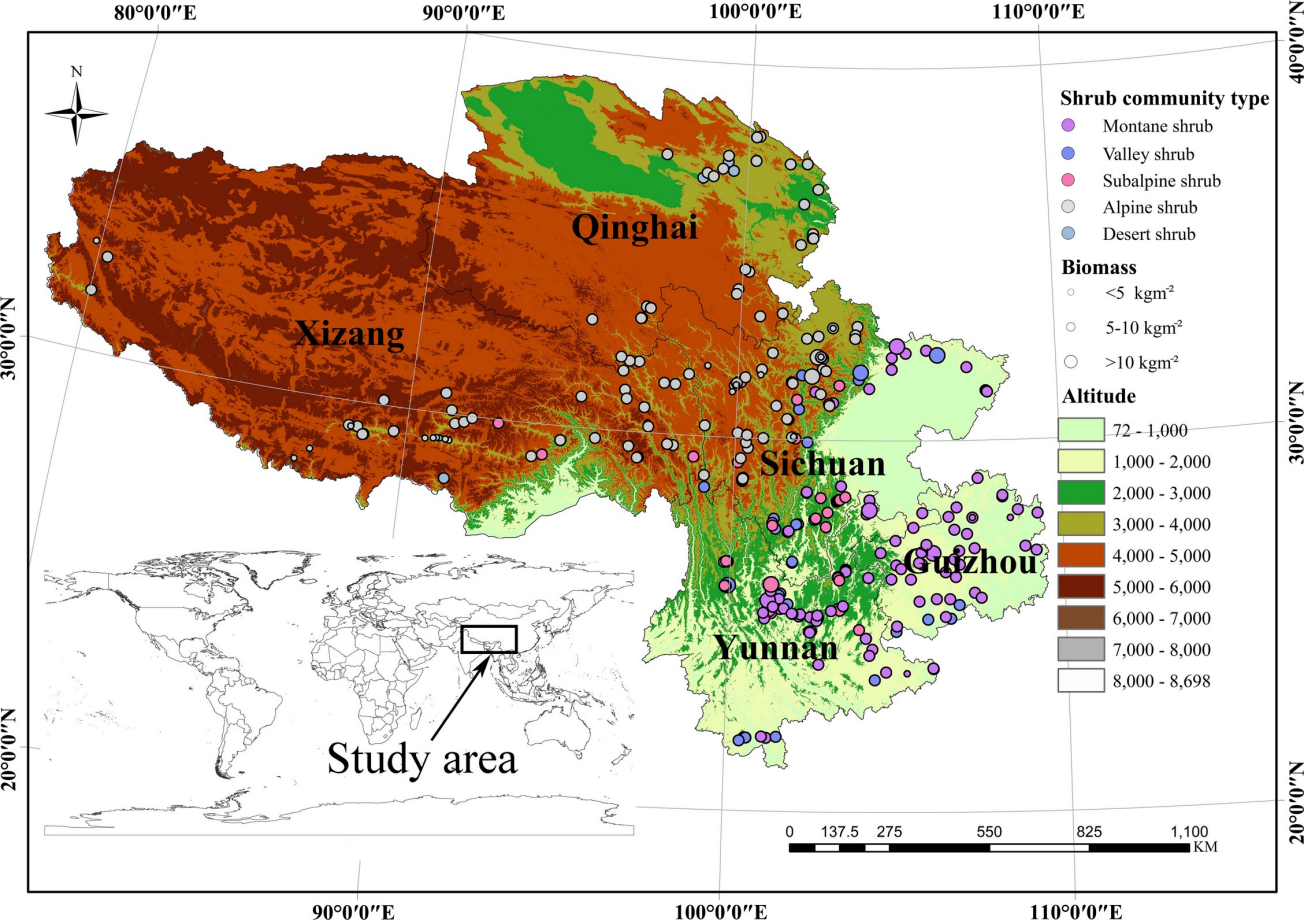
Study area, sampling sites, shrub community type and the spatial distribution of total biomass (TB) at community level of mountainous shrubs in Southwest China.

The study region (21.88° to 38.09° N and 79.85° to 109.25° E) including five provinces in China, covers an area of 2.97×10^6^ km^2^ and encompasses an extensive altitudinal gradient (Fig 1). The MAT and MAP are from -4.8 to 21.7°C and 94.1 to 1630.9 mm, respectively, in this region (S1 Table). Detailed information of 331 sample sites, shrub community types, and major dominant species is shown in Fig 1 and Table 1. The sample sites contain five shrub communities (i.e., valley shrub, montane shrub, subalpine shrub, alpine shrub, and desert shrub) [36]. As shown in Table 1, the major dominant species are *Vaccinium bracteatum* Thunb., *Myrica nana* A. Cheval., *Pyracantha fortuneana* (Maxim.) Li, and *Quercus fabri* Hance in montane shrub community; *Phyllanthus emblica* Linn., *Bauhinia brachycarpa* var. *microphylla* (Oliv. ex Craib) K. et S. S. Larsen, and *Cotinus coggygria* Scop. in valley shrub community; *Rhododendron telmateium* Balf. f. & W. W. Sm., *Sophora moorcroftiana* (Benth.) Baker, *R*. *nivale* Hook. f., *Sibiraea angustata* (Rehd.) Hand.-Mazz., *Potentilla fruticosa* L., *Salix oritrepha* Schneid., and *Thymus mongolicus* Ronn. in alpine shrub community; *R*. *racemosum* Franch. and *Quercus monimotricha* Hand.-Mazz. in subalpine shrub community; and *Myricaria Squamosa* Desv., *Nitraria tangutorum* Bobrov, *and Ephedra gerardiana* Wall. in desert shrub community.

**Table 1:** Geographical information, major species and distribution area at 5 elevation degrees of mountainous shrubs in Southwest China.

Altitude (m)	*n*	Distribution area (province)	Latitude (°N)	Longitude (°E)	Major shrub communities	Major dominant species
331–1000	36	Guizhou, Sichuan, Yunnan	21.97–32.73	101.20–109.25	montane shrub	*Vitex negundo*, *Pyracantha fortuneana*, *Quercus fabri*, *Rhus chinensis*, *Coriaria nepalensis*, *Loropetalum chinense*, *Opuntia stricta*, *Phyllanthus emblica*
1000–2000	72	Guizhou, Yunnan, Sichuan	21.88–32.54	100.43–108.20	montane shrub, valley shrub	*Q*. *fabri*, *P*. *fortuneana*, *Phyllanthus emblica*, *Zanthoxylum armatum*, *Myrica nana*, *Vaccinium bracteatum*, *Bauhinia brachycarpa*, *C*. *nepalensis*
2000–3000	65	Yunnan, Sichuan, Qinghai, Xizang, Guizhou	24.67–38.09	93.82–104.50	montane shrub, subalpine shrub	*V*. *bracteatum*, *M*. *nana*, *Q*. *monimotricha*, *Rhododendron adenogynum*, *R*. *racemosum*, *Rosa sericea*, *Corylus yunnanensis*, *Pinus yunnanensis*
3000–4000	88	Sichuan, Qinghai, Xizang, Yunnan	25.99–38.06	88.28–103.73	alpine shrub, subalpine shrub, desert shrub	*Sophora moorcroftiana*, *Sibiraea angustata*, *R*. *thymifolium*, *R*. *telmateium*, *Potentilla fruticosa*, *Salix oritrepha*, *Spiraea myrtilloides*, *R*. *adenogynum*, *R*. *racemosum*, *P*. *parvifolia*, *Q*. *aquifolioides*, *R*. *sericea*, *M*. *squamosa*, *Nitraria tangutorum*, *Ephedra gerardiana*
4000–5000	70	Xizang, Sichuan, Qinghai	28.30–34.51	79.85–102.89	alpine shrub	*R*. *nivale*, *R*. *telmateium*, *R*. *thymifolium*, *S*. *oritrepha*, *P*. *fruticosa*, *Sabina pingii*, *S*. *angustata*, *Caragana versicolor*, *S*. *sclerophylla*

### Field biomass survey

Shrubs are defined as medium-sized woody or small plants and are distinguished from trees by their short height (below 5 m) and multiple stems [37]. The patchy distribution and large differences in shrub size lead to large within-stand variations in biomass. Some stands have scattered clumps of shrubs, while other stands have a dense and uniform shrub layer in our study area [38]. Therefore, we divided shrubs into three types (i.e., forest-like shrubs (A), grass-like shrubs (B) and xeric shrubs (C)) to investigate the biomass. Type A represented shrub branches with clear, countable stems, such as *Rhododendron*. Type B represented shrub branches with unclear, uncountable stems, such as *Lespedeza bicolor*, or branches with clear and countable but unmeasurable stems, such as *Caragana versicolor*. Type C represented shrubs without branches or those with plexiform growth, such as *Nanophyton erinaceum*.

We sampled 331 sites with shrub coverage greater than 30% across Southwest China during the growing seasons (from July to August) of 2011–2013. At each site, three plots (5 m × 5 m) were randomly established, except for desert shrub where the plots were 10 m × 10 m because of the sparse species distribution and low biomass in desert shrub ecosystems. The distances among different plots were 5–50 m. For the A and C shrub types, 3–5 individuals or clusters of each species with different heights and crown diameter sizes were selected as standard individuals/clusters and harvested to measure AGB and BGB in each plot. More than 30 standard individuals/clusters in total for each species were collected to construct allometric models. Then, the AGB, BGB and TB (total biomass) of these shrub species were deduced from these allometric models based on the determined heights and crown diameters [17, 21, 36]. For the B shrub type and the herb layer, we set three subplots (1 m × 1 m) in each plot, and all B shrubs and herbs in the subplot were harvested to measure AGB and BGB [17, 21]. The roots of shrubs and herbs were excavated to the maximum root depth in the corresponding subplots to determine BGB. Root samples were washed free of soil and separated into shrub and herb roots according to the colour and morphological characters [21]. Shoot and root biomasses were oven dried at 65°C to constant weight and weighed to the nearest 0.1 g.

### Geographic and climatic factors

The geographic information (longitude, latitude, and altitude) of the sample sites was recorded using a global positioning system (GPS). MAP and MAT were obtained for each site from the China Meteorological Forcing Dataset with a spatial resolution of 0.1° × 0.1° in latitude and longitude and every 3 h from 1981 to 2008 [37, 39, 40]. Considering the varied and complicated topography and different drought conditions in Southwest China, we also analysed how the biomass changed with the drought index (reconnaissance drought index, RDI). The RDI is the P (precipitation)/PET (potential evapotranspiration) and was assessed via the computation of PET based on the Thornthwaite method using DrinC software [37, 41–43]. A positive RDI value indicates a wet period, while a negative value represents a dry period. The severity of drought can be divided into four classes according to the RDI: mild (-0.5 to -1.0), moderate (-1.0 to -1.5), severe (-1.5 to -2.0) and extreme (< -2.0) [37, 44].

### Data analysis

In this study, we measured the biomass and its components in 331 shrublands (993 plots) along an extensive altitudinal gradient (311–4911 m) in Southwest China. Plant community varies along altitude. Broad-leaved forest is distributed at 0–1000 m, the valley shrub is at 1000–2000 m, coniferous forest is at 2000–3000 m, alpine grassland is at 3000–4000 m, and alpine scree is at 4000–5000 m [45]. Therefore, according to a previous study [45], the sampling sites were divided into five groups according to their altitudes: I, 0–1000 m; II, 1000–2000 m; III, 2000–3000 m; IV, 3000–4000 m; V, 4000–5000 m (Table 1).

The effects of altitude on the biomass accumulation and allocation of the shrub community were tested by one-way ANOVA, and Tukey’s test was used for multiple comparisons among the five altitude groups. The BGB and biomass allocation proportion of the shrub layer; the BGB, TB and biomass allocation proportion of the shrub community; and the AGB, BGB, TB and biomass allocation proportion of the herb layer showed no homogeneity of variances after log_10_-transformation. Therefore, the Kruskal-Wallis test (nonparametric) was used followed by pair-wise comparisons to test the variations in these parameters among altitude groups. The relationship between biomass and climatic factors was tested by redundancy analysis (RDA) and Pearson correlation analysis. Linear and nonlinear regressions were used to indicate the different trends of biomass and the R/S along climatic gradients. All statistical tests were considered significant at the 0.05 level.

The AGB and BGB partitioning patterns were analysed by using log AGB = α log BGB+log β [25]. The isometric allocation hypothesis suggests that the slope (α) of this equation is not significantly different from 1.0 and that there are no significant changes with environmental conditions [3, 19, 21, 22]. In contrast, the optimal partitioning (allometric) hypothesis shows that the slope (α) is significantly different from 1.0, and plants respond to variations in environmental conditions by altering their biomass allocation among various organs to capture light, water and nutrients to maximize their growth rate [3, 21, 46, 47]. The relationship between the log-transformed AGB and BGB was tested by ordinary least squares (OLS) and standardized major axis (SMA) analyses. OLS analyses were used to establish regression models [23]. SMA analyses were used to establish and evaluate allometric models (log y = log β+α log χ) for above- and below-ground relationships [19, 48]. The confidence intervals (95%) of the slope (α) and y-intercept (log β), calculation of common slopes, and test for homogeneity of slopes were determined by the software package ‘Standardized Major Axis Tests and Routines’ Version 2.0 (http://www. bio.mq.edu.au/ ecology/SMATR/) [49].

The statistical analyses were conducted using SPSS version 20.0 (SPSS Inc., Chicago, IL, USA), and the RDA was performed using the ‘vegan’ package of R [50]. The figures were created using Origin 9.0 (Origin Lab, Northampton, Massachusetts, USA, www.OriginLab.com).

## Results

### 1. Biomass accumulation and allocation along the altitudinal gradient

**Fig 2:**
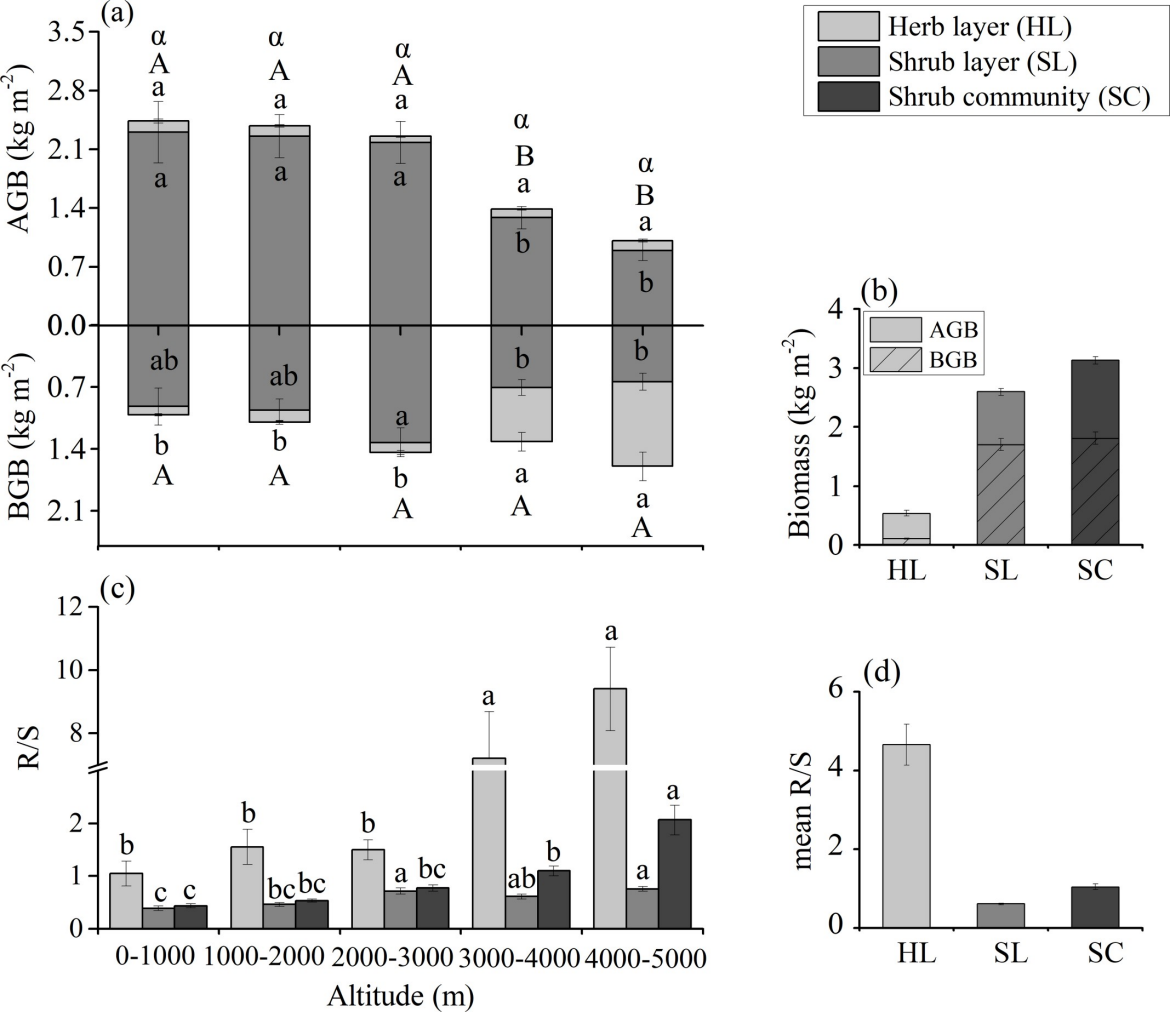
Biomass and mean R/S allocation among five altitude groups. (a) Above-ground biomass (AGB) and below-ground biomass (BGB) allocation among five altitudes and mean biomass of herb layer (HL), shrub layer (SL) and shrub community (SC) in all sites. (b) Mean R/S allocation among five altitude groups.

**Fig 3:**
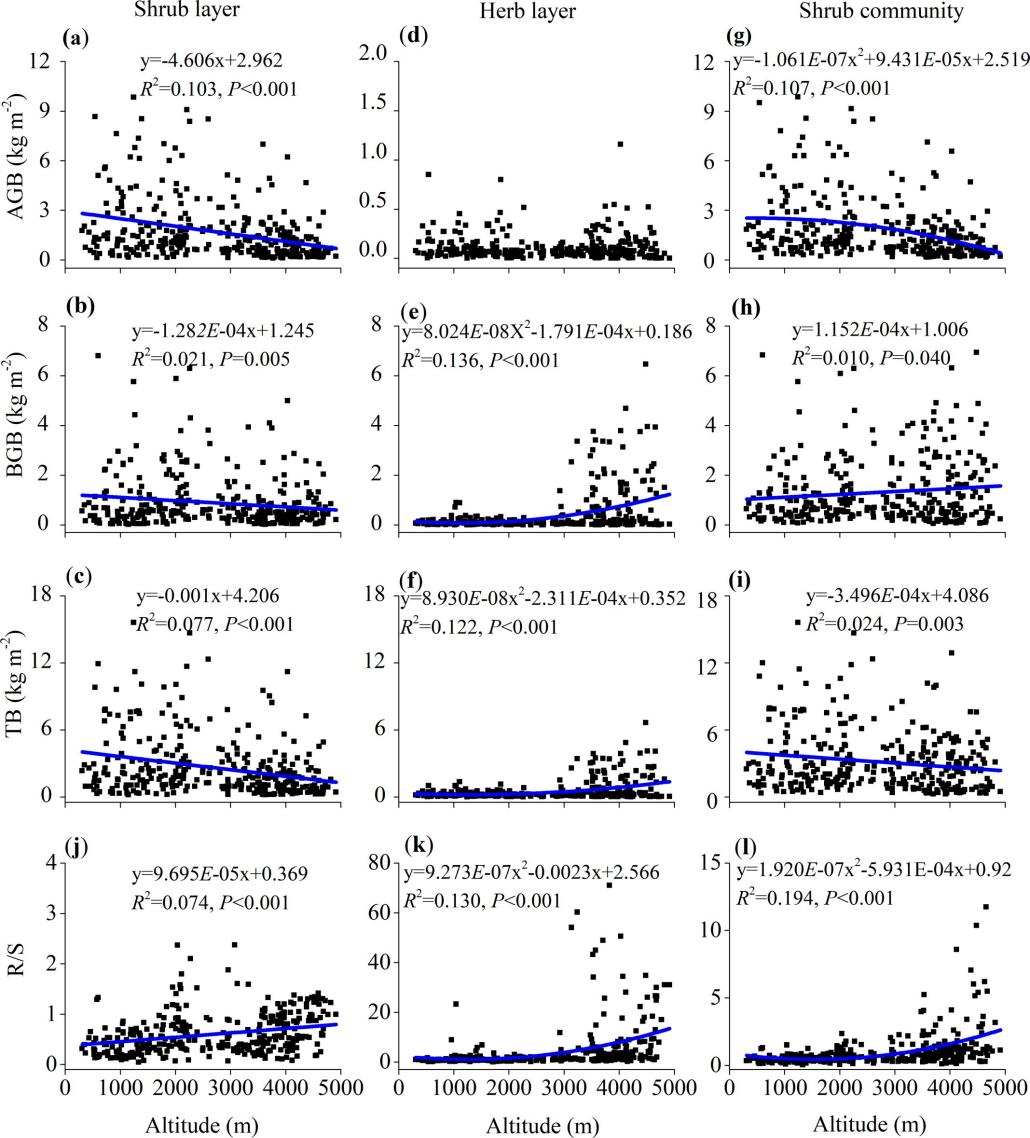
The regression relationship among biomass and R/S with altitude. The regression relationship among above-ground biomass (AGB), below-ground biomass (BGB), and total biomass (TB) of shrub layer (a, b, c, respectively), herb layer (d, e, f, respectively) and shrub community (g, h, i, respectively) with altitude; the regression relationship between R/S of shrub layer (j), herb layer (k) and shrub community (l) and altitude.

The mean AGB and TB of the whole shrub community decreased with increasing altitude, ranging from 1.01 to 2.44 kg·m^-2^ and 2.61 to 3.69 kg·m^-2^, respectively (Fig 2A). The mean community-level TB and AGB at 3000–5000 m decreased by 24.95% and 49.15% compared to those at 0–3000 m, respectively (Fig 2A). The mean AGB, BGB and TB of all investigated sites were 1.81, 1.32, and 3.13 kg·m^-2^, respectively (Fig 2B). The AGB and TB showed a negative quadratic function (R^2^ = 0.107, *P*<0.001, Fig 3G) and linear function (R^2^ = 0.024, *P*<0.01, Fig 3I) with altitude, ranging from 9.86 to 0.15 kg·m^-2^ and 15.61 to 0.26 kg·m^-2^, respectively, but the BGB showed a positive linear function (R^2^ = 0.010, *P*<0.05) with altitude (Fig 3H).

Different lowercase letters indicate significant differences (*P*<0.05) between AGB, BGB and R/S in the shrub layer, herb layer and shrub community among different altitudes. Different capital letters indicate significant differences (*P*<0.05) between AGB and BGB in the shrub community among different altitudes. Different Greek letters indicate significant differences (*P*<0.05) in total biomass (TB) in the shrub community among different altitudes.

The AGB was the most important component of the TB in the shrub layer, and the TB of shrub layer was the most important component (80.31%) of the whole community (Fig 2A, S2 Table). The mean AGB, BGB and TB of shrub layer at all investigative sites were 1.70, 0.89, and 2.59 kg·m^-2^, respectively (Fig 2B). In addition, the mean AGB, BGB and TB were significantly different (*P*<0.05) among different altitude groups, ranging from 0.89 to 2.30 kg·m^-2^, 0.64 to 1.33 kg·m^-2^, and 1.54 to 3.51 kg·m^-2^, respectively (Fig 2A). The mean AGB, BGB and TB of the shrub layer at 3000–5000 m decreased by 51.48%, 37.11% and 46.84% compared to those at 0–3000 m, respectively (Fig 2A). There was a negative linear correlation of AGB, BGB and TB of the shrub layer with increasing altitude, with coefficient R^2^ values of 0.103, 0.021 and 0.077, respectively (*P*<0.01) (Fig 3A, 3B and 3C).

In the herb layer, the mean AGB, BGB and TB of all investigated sites were 0.11, 0.43, and 0.54 kg·m^-2^, respectively (Fig 2B). BGB and TB significantly increased with increasing altitude and reached their maximums at 4000–5000 m, ranging from 0.09 to 0.96 kg·m^-2^ and 0.18 to 1.08 kg·m^-2^, respectively. The mean TB and BGB at 3000–5000 m increased by 2.99- and 5.87-fold compared to those at 0–3000 m, respectively (Fig 2A). The BGB and TB of the herb layer showed a positive quadratic function with altitude (*P*<0.001) (Fig 3E and 3F).

The ratio of AGB/TB in the shrub layer, herb layer and the whole community, as well as the ratio of shrub layer TB to shrub community TB decreased gradually (*P*<0.001) with increasing altitude. Accordingly, the proportion of herb layer TB to the shrub community TB significantly increased with increasing altitude (S2 Table).

The R/S of the shrub layer, herb layer and the community ranged from 0.05 to 2.37, 0.08 to 71.04 and 0.06 to 11.76 across all sites, respectively, and showed positive linear and quadratic functions with increasing altitude (shrub layer, R^2^ = 0.074, *P*<0.001; herb layer, R^2^ = 0.130, *P*<0.001; shrub community, R^2^ = 0.159, *P*<0.001, respectively) (Fig 3J, 3K and 3L). Furthermore, the mean R/S values of the shrub layer, herb layer and shrub community showed significant differences among the five altitude groups, ranging from 0.39 to 0.76 (mean 0.61), 1.05 to 9.41 (mean 4.65), and 0.44 to 2.06 (mean 1.04), respectively (Fig 2C and 2D).

### 2. Allometric relationships between AGB and BGB

**Fig 4:**
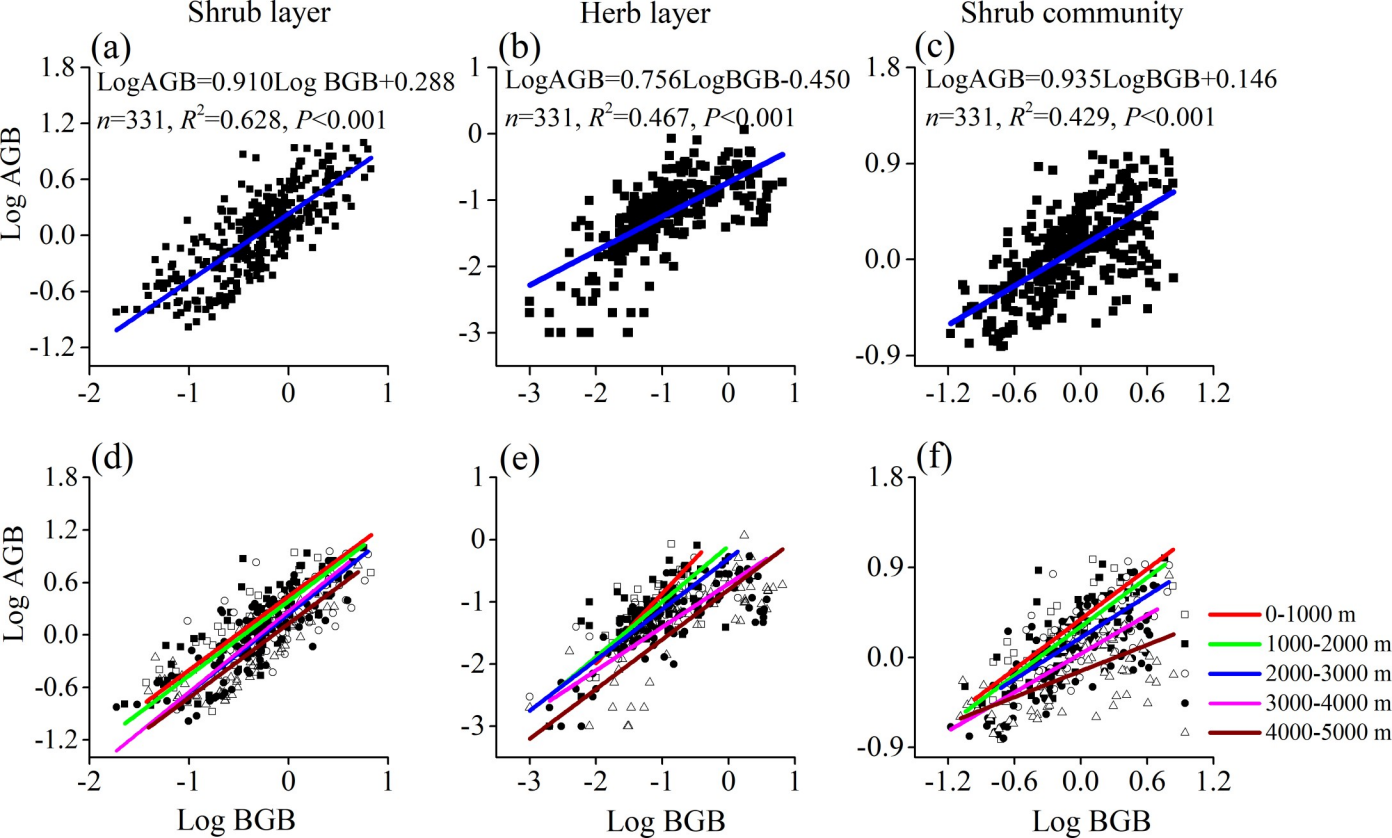
Relationships between above-ground biomass (AGB) and below-ground biomass (BGB) of mountainous shrubs in Southwest China. (a) The slope of the relationship between log AGB and log BGB for the overall shrub layer was 0.91, with 95% confidence intervals of 0.85–0.97. (b) The slope of the relationship between log AGB and log BGB for the overall herb layer was 0.76, with 95% confidence intervals of 0.70–0.82. (c) The slope of the relationship between log AGB and log BGB for the overall shrub community was 0.94, with 95% confidence intervals of 0.86–1.02. The slope of the relationship between log AGB and log BGB for five altitude groups of shrub layer (d), herb layer (e), and shrub community (f) (for detailed slope and 95% confidence intervals, see S3 Table).

The slope (α) of the allometric relationship between log BGB and log AGB for the shrub layer and herb layer at all sites was 0.91 and 0.76, with 95% confidence intervals of 0.85–0.97 and 0.70–0.82, respectively (Fig 4A and 4B, S3 Table), which were significantly different from the isometric relationships (test of isometry, *P*<0.01). The slope (α) of the allometric relationship of the shrub community was 0.94, with a 95% confidence interval of 0.86–1.02 (test of isometry, *P*>0.05) (Fig 4C, S3 Table), which complied with the isometric hypothesis [3, 22, 23].

At different altitude groups, the relationship of log AGB and log BGB complied with different hypotheses. In the shrub layer, log AGB and log BGB showed allometric relationships at 1000–2000 m and 4000–5000 m (test of isometry, *P*<0.05), and isometric relationships (*P*>0.05) were observed in the remaining altitude groups. Moreover, the relationships between AGB and BGB across the five altitude groups in the shrub layer had a common slope (0.873), and the Wald test indicated a significant shift in elevation and axis (Fig 4D, S3 Table). In the herb layer, log AGB and log BGB showed isometric relationships at 0–1000 m and 1000–2000 m (test of isometry, *P*>0.05) but showed allometric relationships at 2000–5000 m. In the herb layer, there was no common slope and the slopes showed significant differences across the five altitude groups (Fig 4E, S3 Table). In the shrub community, the slopes of the different altitudes showed significant differences (Fig 4F, S3 Table). An allometric relationship was observed at 3000–5000 m, but isometric relationships were observed at the other altitudes between log AGB and log BGB. In addition, the five altitude groups did not exhibit a common slope.

### 3. Relationship between biomass and environmental factors

**Fig 5:**
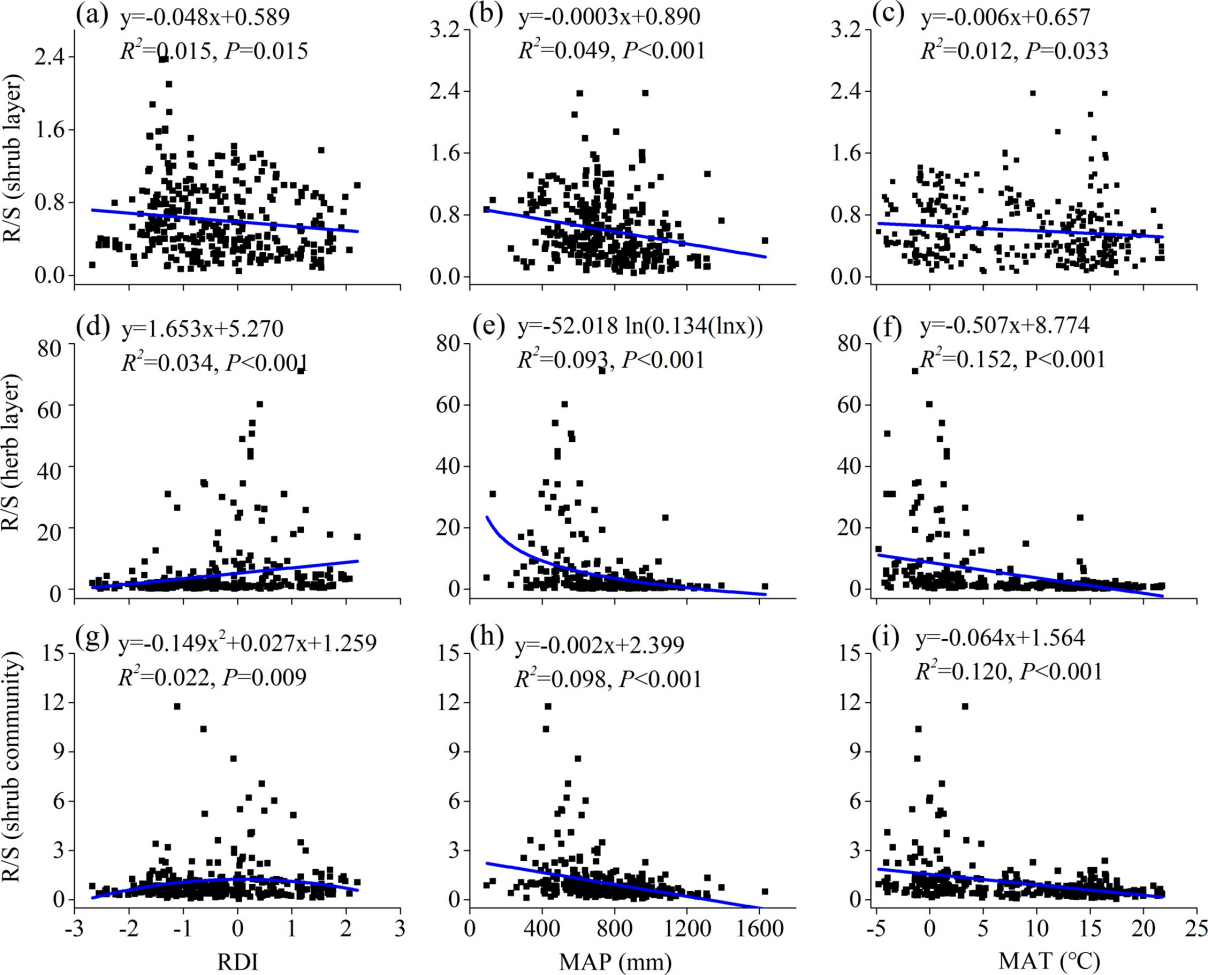
Relationships of R/S with climatic factors. Relationships among R/S with RDI (a), MAP (b), and MAT (c) of the shrub layer; relationships among R/S with RDI (d), MAP (e), and MAT (f) of the herb layer; relationships among R/S with RDI (g), MAP (h), and MAT (i) of the shrub community. R/S, root/shoot ratio; RDI, reconnaissance drought index; MAT, mean annual temperature; MAP, mean annual precipitation.

The Pearson correlation analysis indicated that only BGB and TB of the herb layer were significantly positively related to the RDI (*P*<0.001) (Table 2). The linear regression showed that the increasing RDI was significantly negatively correlated with the R/S of the shrub layer (R^2^ = 0.015, *P*<0.05, Fig 5A), but positively correlated with the R/S of the herb layer (R^2^ = 0.034, *P*<0.001) (Fig 5D). A quadratic function relationship was found between the RDI and the R/S of the shrub community (R^2^ = 0.022, *P<*0.01, Fig 5G).

**Table 2:** Pearson correlation among biomass or biomass ratio with climatic factors.

Synusia	Biomass or biomass ratio	RDI	MAT	MAP
Shrub layer	AGB	-0.056	0.281***	0.266***
	BGB	-0.093	0.158**	0.146**
	TB	-0.075	0.254***	0.239***
Herb layer	AGB	0.045	-0.096	0.225***
	BGB	0.195***	-0.412***	-0.257***
	TB	0.192***	-0.406***	-0.213***
Shrub community	AGB	-0.053	0.280**	**0.280** **
	BGB	-0.056	-0.054**	-0.054
	TB	-0.008	0.162***	0.162**
Herb layer/shrub layer	AGB	0.014	0.027	0.027
	BGB	0.100	-0.190**	-0.190**
	TB	0.096	-0.193***	-0.193***

AGB, above-ground biomass; BGB, below-ground biomass; TB, total biomass; RDI, reconnaissance drought index; MAT, mean annual temperature; MAP, mean annual precipitation.

*, *P*<0.05

**, *P*<0.01

***, *P*<0.001.

Shrub layer biomass, AGB of the herb layer, and AGB and TB of the community were positively related to MAP (*P*<0.01). The BGB and TB of the herb layer and herb layer/shrub layer were negatively correlated with MAP (*P*<0.01) (Table 2). The R/S showed a significantly negative trend with increasing MAP (shrub layer R^2^ = 0.049, *P*<0.001; herb layer R^2^ = 0.093, *P*<0.001; shrub community R^2^ = 0.098, *P*<0.001) (Fig 5B, 5E and 5H).

The shrub layer biomass and AGB of the shrub community were significantly positively related to MAT (*P*<0.01). However, the BGB of the shrub community, BGB and TB of the herb layer and herb layer/shrub layer showed negative correlations with MAT (*P*<0.01) (Table 2). The R/S showed a significantly negative trend with increasing MAT (shrub layer R^2^ = 0.012, *P*<0.05, herb layer R^2^ = 0.152, *P*<0.001, shrub community R^2^ = 0.120, *P*<0.001) (Fig 5C, 5F and 5I). The RDA results showed that altitude, MAT, MAP and the RDI all significantly affected biomass allocation (S1 Fig).

## Discussion

### 1. Variations in biomass accumulation and allocation with altitude

The overall average AGB (1.80 kg·m^-2^) at the community level in this study was higher than that in grass/shrub savanna in the Amazon basin [51] but was lower than that in global sclerophyllous shrubs [52]. The TB of shrublands in Southwest China (3.13 kg·m^-2^) was higher than that in northern Minnesota (1.12 kg·m^-2^) [38]. The difference likely exists because that the plant compositions of the investigated shrub communities are different as a result of the different climate and other environmental factors. For example, the coverage and biomass accumulation of the plant (especially for shrub) in grass/shrub savanna with harsh environmental conditions were much lower than those in alpine shrub land [16, 21, 51]. Similar to this study, the mean AGB and TB of the whole community and shrub layer were also decreased with increasing altitude, since climate conditions for plant growth worsens with the increasing altitude [53, 54].

The average R/S in this study (0.75) was also lower than that in global sclerophyllous shrubs (1.2) [52]. According to the optimal partitioning hypothesis, plants invest much more biomass in shoots in order to enhance photosynthesis under favourable environmental conditions such as humidity, temperature, nutrients, ect., but allocate more biomass to roots under unfavourable conditions [3, 46, 47, 55, 56]. Mountainous shrubs in Southwest China are distributed in the relatively warm and humid regions [16]. Thus, the favourable temperature and precipitation may lead to much more biomass allocation to above-ground organs and consequently cause a decreased R/S in this region. Furthermore, as a result of the decreasing precipitation and temperature (S1 Table), the R/S gradually increased with increasing altitude (Fig 3). These results further confirmed that plants would invest much more biomass in root construction and acquire more resources to defend against the long and cold winters at high altitudes [27, 53, 54, 56, 57].

### 2. Change in allometric relationships

Similar to the relationships between the log-transformed AGB and BGB in forests and grasslands [25, 29], the community-level biomass allocation of mountainous shrubs was supported by the isometric hypothesis in this study (Fig 4C, S3 Table). However, it is interesting to note that the allometric hypothesis fits both the herb layer and shrub layer. The different results between the shrub community and single herb or shrub layer likely account for the offset effects of the two layers. The R/S of the shrub layer was less than 1.0, but the R/S of the herb layer was greater than 1.0, and consequently the R/S of the shrub community was closer to 1.0 (Fig 2B). Moreover, the relationships between AGB and BGB of the five altitude groups were also different. The allometric relationships were observed at 3000–5000 m in the shrub community, 4000–5000 m in the shrub layer and 2000–5000 m in the herb layer in this study. These results are similar to the desert shrub biomass allocation on the Tibetan Plateau [21] and in the harsh Tibetan Plateau alpine steppe ecosystem [2]. Plants will allocate more photosynthate to roots (Figs 2 and 3), so as to obtain more water and nutrients for growth at higher altitudes with decreased precipitation and temperature [53, 54] (S1 Table). On the other hand, similar to the isometric theory of biomass partitioning in alpine shrubs and alpine grasslands in previous studies [21, 29], the shrub community at 0–3000 m, the shrub layer at 0–1000 m, and the herb layer at 0–2000 m all fit the isometric relationship due to the higher moisture and temperature at lower altitudes (S1 Table).

### 3. Effects of climatic factors on biomass partitioning

The R/S values of the shrub layer and shrub community were significantly negatively correlated with the RDI (Fig 5A and 5G), which supports existing hypotheses that the R/S decreases at wet sites as moisture availability increases [1, 58]. As mentioned above, the increased precipitation promoted the accumulation of shoot biomass in shrub ecosystems [51]. AGB of the shrub community and its component synusiae increased with MAP, and consequently the R/S was negative with MAP in this study. These results were similar with those reported for global shrubs and grasslands [1, 59] but opposite to those of the northeast Tibetan Plateau shrubs [21]. In northeast Tibetan Plateau shrubs [21], the MAP was negatively corrected with MAT, therefore, plants would have a high use efficiency of precipitation in BGB due to vegetational and biogeochemical constraints, ultimately leading to an increased R/S at lower temperatures accompanied by increasing precipitation in northeast Tibetan [21]. However, MAP was positively correlated with MAT (R = 0.522, *P*<0.01) in this study. Therefore, warmer temperature with increasing precipitation may asymmetrically enhance the growth of shoots and decrease the R/S [1, 9, 58]. The biomass of the shrub community and shrub layer decreased with decreasing MAT as a result of cold temperatures directly reducing plant photosynthesis and indirectly limiting microbial activity [31], inhibiting the decomposition and mineralization of organic matter, and leading to a reduction in plant growth and productivity [10, 60]. In addition, the temperature increases by 0.56°C per 100 m decrease in altitude [61], and the MAT and MAP are higher at lower altitudes (S1 Table). The shrub communities were mainly montane shrub, valley shrub and subalpine shrub at lower altitudes [36] with higher temperature (S1 Table); these shrub communities can accumulate more biomass than alpine shrubs and desert shrubs in lower temperature regions at high altitudes. Usually, plants allocate much more biomass to roots for nutrient absorption, exhibit slower root turnover, and accumulate much more root biomass in cold regions [10, 60]. Consistent with the significant decreasing trend of the R/S with increasing temperature in grasslands [24, 59] and shrubs [1, 21], the R/S in both shrub and herb layer increased significantly with decreased MAT in this study.

## Conclusion

This study describes the large-scale patterns of biomass accumulation and allocation of mountainous shrubs with altitude and their relationships with climatic variables in Southwest China. The total biomass of the shrub community and shrub layer decreased with increasing altitude; however, biomass accumulation of the herb layer was increased at higher altitudes in this region. The R/S of the whole community and component synusia increased gradually with increasing altitudes (*P*<0.001) as a result of lower MAP and MAT. The allometric relationship between AGB and BGB was found for the shrub and herb layer, but an isometric relationship was found for the whole shrub community because of the offset effects of the two layers. These findings indicate that mountainous shrub biomass accumulation and allocation are strongly related to altitude and are significantly affected by MAP and MAT in Southwest China.

## Supporting information

S1 Fig RDA ordination of above-ground biomass (AGB), below-ground biomass (BGB), total biomass (TB) and root/shoot ratio (R/S) of the shrub layer (SL), herb layer (HL) and shrub community (SC) with climatic factors (mean annual temperature (MAT), mean annual precipitation (MAP), and reconnaissance drought index (RDI)) and geographic (longitude, latitude, altitude) factors. (DOCX)

S1 Table Comparison of climatic factors among five altitude groups (mean ± standard error) of mountainous shrubs in Southwest China. (DOCX)

S2 Table The biomass allocation proportion of mountainous shrubs at different altitudes in Southwest China (mean ± standard error). (DOCX)

S3 Table Allometric scaling exponents and the test of isometry between log AGB and log BGB of mountainous shrubs in Southwest China. (DOC)
