# Supplementary material for: Altitudinal pattern of shrub biomass allocation in Southwest China
Source: PLoS One. 2020 Oct 22;15(10):e0240861. doi: 10.1371/journal.pone.0240861 (PMC7580895; doi:10.1371/journal.pone.0240861)
Supplement: S1 Fig — (DOCX) [file pone.0240861.s001.docx]

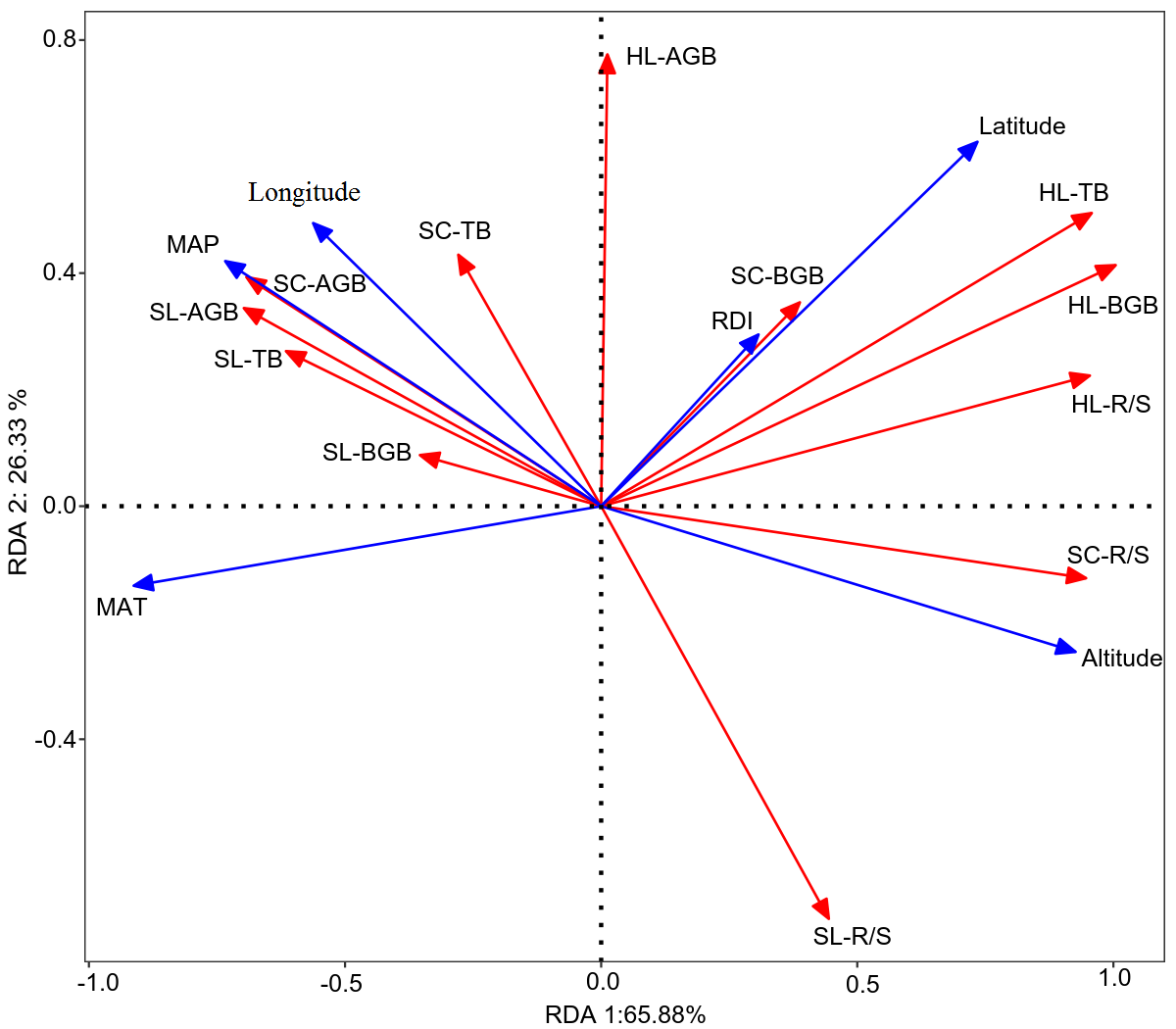


**S1 Fig. RDA ordination of above-ground biomass (AGB), below-ground biomass (BGB), total biomass (TB) and root/shoot ratio (R/S) of the shrub layer (SL), herb layer (HL) and shrub community (SC) with climatic factors (mean annual temperature (MAT), mean annual precipitation (MAP), and reconnaissance drought index (RDI)) and geographic (longitude, latitude, and altitude) factors.**
