# Supplementary material for: Altitudinal pattern of shrub biomass allocation in Southwest China
Source: PLoS One. 2020 Oct 22;15(10):e0240861. doi: 10.1371/journal.pone.0240861 (PMC7580895; doi:10.1371/journal.pone.0240861)
Supplement: S1 Table — (DOCX) [file pone.0240861.s002.docx]

**S1 Table. Comparison of climatic factors among five altitude groups (mean ± standard error) of mountainous shrubs in Southwest China.**

| **Altitude (m)** | ***n*** | **MAT (℃ )** | | **MAP (mm)** | | **RDI** | |
| --- | --- | --- | --- | --- | --- | --- | --- |
|  |  | **mean** | **range** | **mean** | **range** | **mean** | **range** |
| 0-1000 | 36 | 15.86±0.54 a | 2.94-21.68 | 1060.71±29.41 a | 761.85-1389.96 | -0.28±0.14 bc | -2.01-1.71 |
| 1000-2000 | 72 | 14.73±0.48 a | -3.62-21.75 | 893.25±21.89 b | 555.32-1630.98 | -0.72±0.12 cd | -2.67-1.51 |
| 2000-3000 | 65 | 11.72±0.56 b | -0.98-17.52 | 718.33±17.39 c | 228.00-953.97 | -1.20±0.09 d | -2.55-1.38 |
| 3000-4000 | 88 | 3.32±0.49 c | -4.22-14.27 | 626.86±20.76 d | 259.06-969.92 | -0.06±0.12 ab | -2.43-2.06 |
| 4000-5000 | 70 | 0.05±0.24 d | -4.78-4.84 | 599.94±19.82 d | 94.15-937.72 | 0.31±0.13 a | -1.51-2.21 |
| 0-5000 | 331 | 8.12±0.40 | -4.78-21.75 | 744.26±12.81 | 94.15-1630.98 | -0.37±0.06 | -2.67-2.21 |

Different lowercase letters mean the significant differences at *P*＜0.001. MAT, mean annual temperature; MAP, mean annual precipitation; RDI, Reconnaissance Drought Index.
