# Supplementary material for: Altitudinal pattern of shrub biomass allocation in Southwest China
Source: PLoS One. 2020 Oct 22;15(10):e0240861. doi: 10.1371/journal.pone.0240861 (PMC7580895; doi:10.1371/journal.pone.0240861)
Supplement: S2 Table — (DOCX) [file pone.0240861.s003.docx]

**S2 Table. The biomass allocation proportion of mountainous shrubs at different altitudes in Southwest China (mean ±standard error).**

| **Altitude (m)** | **0-1000** | **1000-2000** | **2000-3000** | **3000-4000** | **4000-5000** | **0-5000** |
| --- | --- | --- | --- | --- | --- | --- |
| AGB/TB in SL (%) | 74.05±1.96 a | 70.69±1.49 ab | 62.24±1.80 c | 65.26±1.47 bc | 59.77±1.63 c | 65.64±0.78 |
| AGB/TB in HL (%) | 56.08±2.42 a | 51.12±2.11ab | 45.85±1.73 b | 30.89±1.92 c | 22.76±2.10 c | 39.25±1.13 |
| AGB/TB in SC (%) | 71.24±1.72 a | 67.00±1.29 ab | 60.00±1.73 bc | 54.00±1.75 c | 44.74±2.32 d | 57.92±0.96 |
| HLTB/SLTB (%) | 18.78±4.73 b | 28.69±8.77 b | 11.21±2.83 b | 58.74±10.19 b | 140.71±29.19 a | 55.86±7.47 |
| HLTB/SCTB (%) | 12.65 ±2.32 c | 14.10 ±2.04 bc | 8.04 ±1.31 c | 24.12 ±2.54 ab | 34.30 ±3.58 a | 19.69 ±1.27 |
| SLTB/SCTB (%) | 87.35 ±2.32 a | 85.90 ±2.04 ab | 91.96 ±1.31 a | 75.88 ±2.54 bc | 65.70±3.58 c | 80.31 ±1.27 |

Different lowercase letters mean the significant differences between different altitudes at *P*＜0.001. AGB, above-ground biomass; TB, total biomass; SL, shrub layer; HL, herb layer; SC, shrub community.
