# Supplementary material for: Altitudinal pattern of shrub biomass allocation in Southwest China
Source: PLoS One. 2020 Oct 22;15(10):e0240861. doi: 10.1371/journal.pone.0240861 (PMC7580895; doi:10.1371/journal.pone.0240861)
Supplement: S3 Table — (DOC) [file pone.0240861.s004.doc]

**S3 Table. Allometric scaling exponents and the test of isometry between log AGB and log BGB of mountainous shrubs in Southwest China.**

| **Synusia** | **Altitude(m)** | ***R^2^*** | ***P*** | **Slope/Common slope (α) （95% confidence interval）** | **Intercept（logβ）(95% confidence interval)** | **Test of isometry** | |
| --- | --- | --- | --- | --- | --- | --- | --- |
|  |  |  |  |  |  | ***F*** | ***P*** |
| Shrub layer | 0-1000 | 0.744 | <0.001 | 0.843 (0.707,1.005) a | 0.440 (0.347,0.534) | 3.894 | **0.057** |
|  | 1000-2000 | 0.666 | <0.001 | 0.850 (0.741,0.975) a | 0.384 (0.313,0.455) | 5.603 | 0.021 |
|  | 2000-3000 | 0.480 | <0.001 | 0.897 (0.749,1.075) a | 0.239 (0.166,0.312) | 1.428 | **0.236** |
|  | 3000-4000 | 0.655 | <0.001 | 0.923 (0.814,1.046) a | 0.271 (0.198,0.345) | 1.623 | **0.206** |
|  | 4000-5000 | 0.635 | <0.001 | 0.845 (0.730,0.977) a | 0.128 (0.048,0.208) | 5.352 | 0.024 |
|  |  |  | 0.853 | 0.873 (0.818,0.933) |  |  |  |
|  | 0-5000 | 0.628 | <0.001 | 0.910 (0.852,0.972） | 0.288 (0.252,0.324) | 7.933 | 0.005 |
| Herb layer | 0-1000 | 0.497 | <0.001 | 1.125 (0.881,1.437) a | 0.261 (-0.084,0.607) | 0.940 | **0.339** |
|  | 1000-2000 | 0.482 | <0.001 | 0.892 (0.752,1.058) ab | -0.101 (-0.295,0.090) | 1.766 | **0.188** |
|  | 2000-3000 | 0.681 | <0.001 | 0.816 (0.709,0.941) bc | -0.302 (-0.457, -0.148) | 8.236 | 0.006 |
|  | 3000-4000 | 0.601 | <0.001 | 0.697 (0.609,0.797) c | -0.708 (-0.820, -0.597) | 29.386 | <0.001 |
|  | 4000-5000 | 0.631 | <0.001 | 0.799 (0.690,0.925) bc | -0.805 (-0.934, -0.677) | 9.488 | 0.003 |
|  | 0-5000 | 0.467 | <0.001 | 0.756 (0.699,0.818) | -0.450 (-0.574, -0.426) | 49.507 | <0.001 |
| Shrub community | 0-1000 | 0.730 | <0.001 | 0.976 (0.815,1.169) a | 0.411 (0.328,0.495) | 0.075 | **0.786** |
|  | 1000-2000 | 0.637 | <0.001 | 1.021 (0.885,1.178) a | 0.333 (0.273,0.393) | 0.081 | **0.777** |
|  | 2000-3000 | 0.475 | <0.001 | 1.011 (0.844,1.213) a | 0.194 (0.124,0.264） | 0.015 | **0.903** |
|  | 3000-4000 | 0.563 | <0.001 | 0.858 (0.745,0.988) a | 0.061 (-0.002,0.124) | 4.654 | 0.034 |
|  | 4000-5000 | 0.390 | <0.001 | 0.697 (0.578,0.842) b | -0.126 (-0.201, -0.051) | 15.118 | <0.001 |
|  | 0-5000 | 0.429 | <0.001 | 0.935 (0.862, 1.015) | 0.146 (0.108,0.184) | 2.596 | **0.108** |
